# Smart Moves: Effects of Relative Brain Size on Establishment Success of Invasive Amphibians and Reptiles

**DOI:** 10.1371/journal.pone.0018277

**Published:** 2011-04-06

**Authors:** Joshua J. Amiel, Reid Tingley, Richard Shine

**Affiliations:** School of Biological Sciences, University of Sydney, Sydney, New South Wales, Australia; Cajal Institute, Consejo Superior de Investigaciones Científicas, Spain

## Abstract

Brain size relative to body size varies considerably among animals, but the ecological consequences of that variation remain poorly understood. Plausibly, larger brains confer increased behavioural flexibility, and an ability to respond to novel challenges. In keeping with that hypothesis, successful invasive species of birds and mammals that flourish after translocation to a new area tend to have larger brains than do unsuccessful invaders. We found the same pattern in ectothermic terrestrial vertebrates. Brain size relative to body size was larger in species of amphibians and reptiles reported to be successful invaders, compared to species that failed to thrive after translocation to new sites. This pattern was found in six of seven global biogeographic realms; the exception (where relatively larger brains did not facilitate invasion success) was Australasia. Establishment success was also higher in amphibian and reptile families with larger relative brain sizes. Future work could usefully explore whether invasion success is differentially associated with enlargement of specific parts of the brain (as predicted by the functional role of the forebrain in promoting behavioural flexibility), or with a general size increase (suggesting that invasion success is facilitated by enhanced perceptual and motor skills, as well as cognitive ability).

## Introduction

The relatively large and complex brain of vertebrates is one of the most characteristic features of this lineage, and is linked to many important features of vertebrate behaviour and ecology. Sophisticated perceptual and cognitive abilities are central to the success of many taxa, and may have imposed powerful selection for increases in relative brain size [1]–[3]. At the same time, however, brains are expensive: on a mass-specific basis, the metabolic cost of brain function is among the highest of any organ [4], [5]. We thus might expect the benefits of increased intellect to be balanced against metabolic costs, with relative brain size in any given species reflecting that tradeoff [2]. How can we test hypotheses about the functional advantages of larger brain size? One way is to argue from design, under the assumption that specific components of the brain have particular functions and that an increase in size of that component will enhance organismal performance in that function [6]–[8]. This method is difficult to apply to overall brain size, however, because of complex correlated shifts in brain structure as well as size [9], [10]. An alternative method, and the one we adopt in the present paper, is to look for correlations between relative brain size and some aspect of ecological functioning.

What kind of challenges should a larger brain help an organism to solve? If cognition is important, a larger-brained individual should be more adept at dealing with novel challenges. High rates of anthropogenic translocation of species around the world [11]–[13] provide an ideal opportunity to test this hypothesis; if a large brain helps to deal with novel challenges, then larger brains should be particularly useful for organisms that are suddenly confronted with a novel set of biotic and abiotic challenges as a result of translocation [3], [10], [14], [15]. Translocated species face a range of novel challenges, such as unfamiliar predators, pathogens, and prey [16]–[18]. Some of those challenges place a premium on an organism's physiology (e.g., thermal tolerance, immune function), but others can be overcome only by organisms that can flexibly modify their behaviour in response to novel cues [19]. In keeping with this hypothesis, species of birds and mammals with larger brain masses relative to body mass tend to have been more successful at establishing viable populations in novel environments [18], [20]. The selective advantage to larger brain size might simply involve more brain tissue (to transmit impulses to and from integrative centres, such as the cerebral cortex: [21]) or an increase in cognitive function (the brain size-environmental change (BS-EC) theory, that larger brains increase behavioural flexibility: [18], [20]).

How general are these results? Amphibians and reptiles have smaller forebrains than do birds and mammals, and are widely believed not to have the same level of behavioural complexity [3], [22]. Plausibly, then, advantages of larger relative brain size may be unique to birds and mammals. Alternatively, a larger brain size might enhance colonizing ability in amphibians and reptiles in the same way as it does in endothermic vertebrates, despite the differences in brain structure between ectotherms and endotherms. A selective advantage to larger brain size in translocated amphibians and reptiles might suggest that a relationship between brain size and the ability to cope with novel conditions reflects broader advantages of increased brain capacity, not just an increase in forebrain (cognitive) function. To examine the generality of the purported relationship between larger brain size and capacity to thrive in a novel environment, we have analysed data on anthropogenic introductions of amphibians and reptiles to areas outside of their native geographic ranges. If a larger brain facilitates dealing with new challenges, we predict that success in establishing viable populations following translocation will be higher in amphibians and reptiles with large brains relative to their body sizes.

## Results

**Figure 1:**
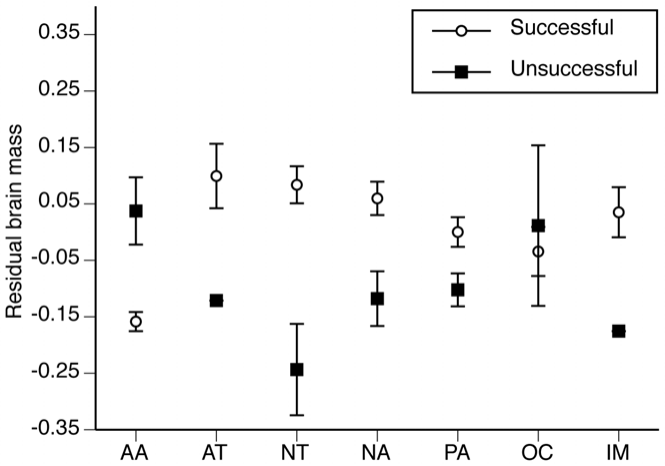
Mean (± SE) residual brain mass of amphibian and reptile species that were successful (open circles) and unsuccessful (dark squares) in establishing populations outside of their native geographic ranges in seven different biogeographic realms. AA  =  Australasia, AT  = Afrotropics, NT  =  Neotropics, NA =  Nearctic, PA  =  Palearctic, OC  =  Oceania, and IM  = Indomalaysia. Lack of standard errors in the AT and IM realms reflect low numbers of unsuccessful introductions.

Overall, patterns of establishment success in translocated amphibians and reptiles support the prediction that species with larger relative brain sizes will be more successful when confronted with environmental change (figure 1). Our analyses of anthropogenic introductions revealed that the probability of successful establishment in a novel environment increased with increasing residual brain mass in six out of seven biogeographic realms. However, the intercept and slope of the relationship between residual brain mass and establishment probability varied according to biogeographic realm (likelihood ratio test between models with and without random slopes: *D* = 8.0, *P* = 0.018). After accounting for taxonomic autocorrelations and propagule pressure, effects of residual brain mass on establishment success were positive in the Palearctic (per-realm intercept ± slope  =  −0.95+0.28), the Nearctic (−0.67+0.65), the Neotropics (1.1+3.0), Indomalaysia (0.73+2.6), Oceania (−0.15+1.36), and the Afrotropics (1.3+3.3), but negative in Australasia (−2.2–1.4). However, 95% prediction intervals on these random intercepts and slopes overlapped zero in Oceania, Indomalaysia, and the Afrotropics (see Figure S1). In the latter two realms, this result was likely due to low recorded numbers of unsuccessful introductions (n = 1 and n = 2, respectively). Omission of these two realms did not influence our finding that establishment success increases with residual brain mass in all realms except Australasia (likelihood ratio test between models with and without random slopes: *D* = 6.7, *P* = 0.035). We also found no evidence to suggest that effects of propagule pressure varied by realm (likelihood ratio test between models with and without random slopes: *D* = 3.6, *P* = 0.17).

**Figure 2:**
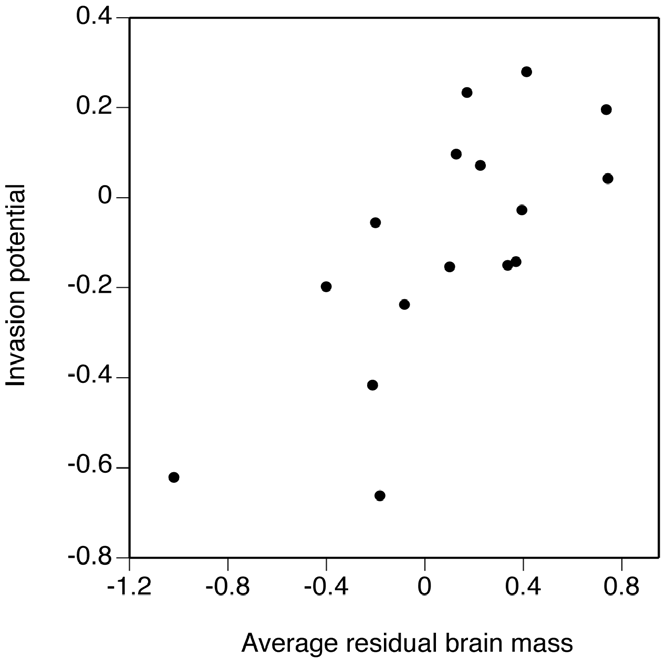
Invasion potential of amphibian and reptile families *versus* mean residual brain mass of each family. See Methods for calculation of invasion potential.

Similar results were obtained for the effects of residual brain mass on invasion success at the family level (figure 2). After accounting for order membership, invasion potential increased with increasing average residual brain mass per family (estimate ± se = 1.2±0.29 in log-log space; *n* = 16, *P* = 0.0022).

## Discussion

Among the species of amphibians and reptiles that have undergone human translocations, those with larger relative brain size have been more successful than smaller-brained species at establishing populations in novel environments. This pattern is relatively consistent in our data, being seen at the familial level, as well as within six of seven biogeographic realms at the species level. The same evolutionary trend is seen in birds and mammals [18], [20], suggesting that larger brain size enhances the ability to deal with novel environmental challenges in all four major classes of terrestrial vertebrates.

Why is a larger relative brain size associated with higher colonization success following translocation? Although the consistency of the correlation taxonomically and geographically suggests a causal connection, the nature of any functional benefits conferred by a larger brain remains unclear. In our analyses, larger brains did not enhance establishment success of translocated ectotherms in all environments. Translocated amphibians and reptiles with smaller (rather than larger) brains were more successful at establishing populations in Australasia. Environmental factors may select against larger brain size if a lack of resources exacerbates the energetic costs of maintaining such an expensive organ. Low resource availability in Australasia may favour phenotypic traits (such as small brain size) that reduce an animal's total energy requirements [23]. Evolutionary trends towards reduced fecundity levels in rodents and in birds that have invaded Australia over longer (evolutionary) time periods accord with this hypothesis [24], [25]. What functional advantages to larger brain size in a novel environment might be strong enough to offset the cost of maintaining a larger brain?

Previous studies on endothermic vertebrates have attributed the relationship between brain size and establishment success to cognitive abilities, in turn linked to the elaboration of forebrain size and capacity in larger-brained mammals and birds [18], [20]. Amphibians and reptiles do not have brain structures directly analogous to the forebrain of birds and mammals, suggesting that an increase in relative brain size is unlikely to confer the same cognitive advantages as would a relatively large brain in a bird or a mammal [3], [9], [10]. There may well be superior cognitive ability in larger-brained amphibians and reptiles, but increases in non-cognitive functions (involving sensory and motor functions, for example) also may have facilitated the survival of vertebrates in novel environments.

Our data do not enable us to discriminate between the alternative explanations for the correlation between brain size and invasion success. Invaders may prosper in novel environments either because of enhanced cognitive skills (presumably related to forebrain size) or to a wider suite of information-processing abilities (related to several parts of the brain). Even if the actual advantage was entirely driven by forebrain size, overall brain size may be highly correlated with absolute forebrain size; and much of the interspecific variation in cognitive ability thus may be driven by variation in overall brain size not in relative importance of the forebrain versus other components. Larger brain size also may increase the level of neural connectivity between brain compartments, thus enhancing the coordination of multiple functions (as in visuomotor relays: [26]). Thus, data on the ecological correlates of overall brain size cannot reveal which brain compartments are functionally significant to animals in novel environments.

To tease apart the functional basis for a relationship between brain size and survival in novel environments, we need to examine how variation in specific brain features (overall size vs. size of individual components vs. density of neural relays) maps onto ecological parameters such as invasion success. For example, a larger medial cortex may confer better memory in reptiles, increasing spatial learning and the ability to locate critical resources in unfamiliar surroundings [27]. Correlative studies of brain size need to include known morphological predictors of brain size as well as geographic and taxonomic variables to give a robust and clear view of brain function and evolution [28].

To minimize confounding factors that are inevitable in any interspecific comparison, research on this topic might usefully focus on geographically wide-ranging species that extend across environments posing a range of challenges to information-processing. An extensive literature on reptiles and amphibians, as well as other taxa, shows that a wide range of morphological, physiological, behavioural and ecological traits can vary considerably across a species' distribution [29]–[31]. Such variation hints that brain size and structure may vary also, providing an exciting opportunity for future work to tease apart the ways in which the characteristics of an animal's brain influences that organism's ability to cope with the challenges posed by both ancestral and novel environmental conditions. Given widespread predictions of substantial changes in abiotic conditions over the range of most species within the next several decades [32], an ability to cope with novel challenges may well prove to be one of the most significant predictors of species viability in the face of global change.

## Materials and Methods

We used data on the success or failure of amphibian and reptile introductions collated by Kraus [13]. Introductions were considered successful if they resulted in the establishment of a viable population according to the most recent literature citation [13]. Following the method used by Sol *et al*. [18], [20], we classified multiple introductions of a single species to an area as one introduction event. Introduction locations consisted of countries, islands, archipelagos, states, or provinces [33]. Data on brain and body mass (*n* = 149 species) were collected from various sources [34]–[38]. Nearly half (*n* = 72) of the species for which we obtained brain-mass data have been introduced outside of their native geographic ranges at least once, providing data on 561 introduction events for our analyses (Amphibia *n* = 229, Reptilia *n* = 332). This ratio of species to introduction events (0.13) is similar to that used in a previous test of differential success due to relative brain size among mammals (0.15: [18]).

Larger species typically have larger brains (see Figure S2), potentially confounding the influences of body and brain mass on establishment success. To remove this allometric effect, we calculated the residuals from a linear regression of taxonomic order and log-body mass on log-brain mass (*n* = 149 species, *R^2^* = 94%. *P*  =  <0.0001). Some insular mammals have brain masses smaller than those predicted using mainland allometric data [39] but there are no data to test for such effects in amphibians and reptiles. Taxonomic order was included as a covariate to account for potential grade shifts between higher taxa [20], [40], [41].

We tested whether residual brain mass correlated with the probability of successful establishment using generalized linear mixed effects models (logit link, binomial error distribution). In all models, the dependent variable was whether or not an introduction attempt had been successful. Because previous research has shown that the total number of independent introduction attempts (propagule pressure) to a given area is a critical determinant of establishment probability [42], [43], we included propagule pressure for each location in all models investigating the relationship between residual brain mass and establishment success (see [13] and [43] for details). To account for taxonomic biases among introduction events, we included species, genus, family, and order as nested random effects. We also included a random effect describing the biogeographic realm in which introductions occurred to control for clustering of introduction attempts within regions. Residual brain mass and log-propagule pressure were entered into the model as fixed effects. A minimum adequate model of establishment probability was derived by conducting likelihood ratio tests between nested models using a backward sequence of variable removal (α = 0.05). P-values produced by this model selection approach are often conservative (i.e., higher than they should be: [44]).

In another set of analyses, we looked for patterns at the familial level rather than treating each species as a separate entity. For each family represented in our introduction database, we first averaged residual brain mass of the species within that family relative to the overall allometric relationship between brain mass and body mass (based on all species for which we had brain-mass data). We then looked for a relationship between this measure of familial-level average residual brain mass and the invasion potential of each family. To estimate familial-level invasion potential, we extracted the family-level random effects coefficients of a generalized linear mixed effects model where the success or failure of each introduction attempt was the dependent variable, propagule pressure was a fixed effect, and species, genus, family, order, and biogeographic realm were random effects [18]. Finally, after accounting for order membership, we investigated whether familial-level invasion potential was correlated with the mean residual brain mass of each family using a linear mixed effects model. Only families that were represented by at least two species in both the introduction and brain-mass databases were included in this analysis (*n* = 16 families). All statistical analyses were conducted in R^©^ 2.9.0 using the lme4 library (Bates and Maechler 2009; R Development Core Team 2009).

## Supporting Information

Figure S1 **95% prediction intervals on the conditional modes of the random intercepts and slopes of the relationship between residual brain mass and establishment probability in amphibian and reptile species.**
(TIF)

Figure S2 **Log brain mass versus log body mass for all amphibian and reptile species used in this study.** The brain mass versus body mass trend inclusive of all biogeographic realms is shown in the top left panel, followed by the trends for each individual biogeographic realm.
(TIF)
