# Supplementary figures and images for: Smart Moves: Effects of Relative Brain Size on Establishment Success of Invasive Amphibians and Reptiles
Source: PLoS One. 2011 Apr 6;6(4):e18277. doi: 10.1371/journal.pone.0018277 (PMC3071803; doi:10.1371/journal.pone.0018277)

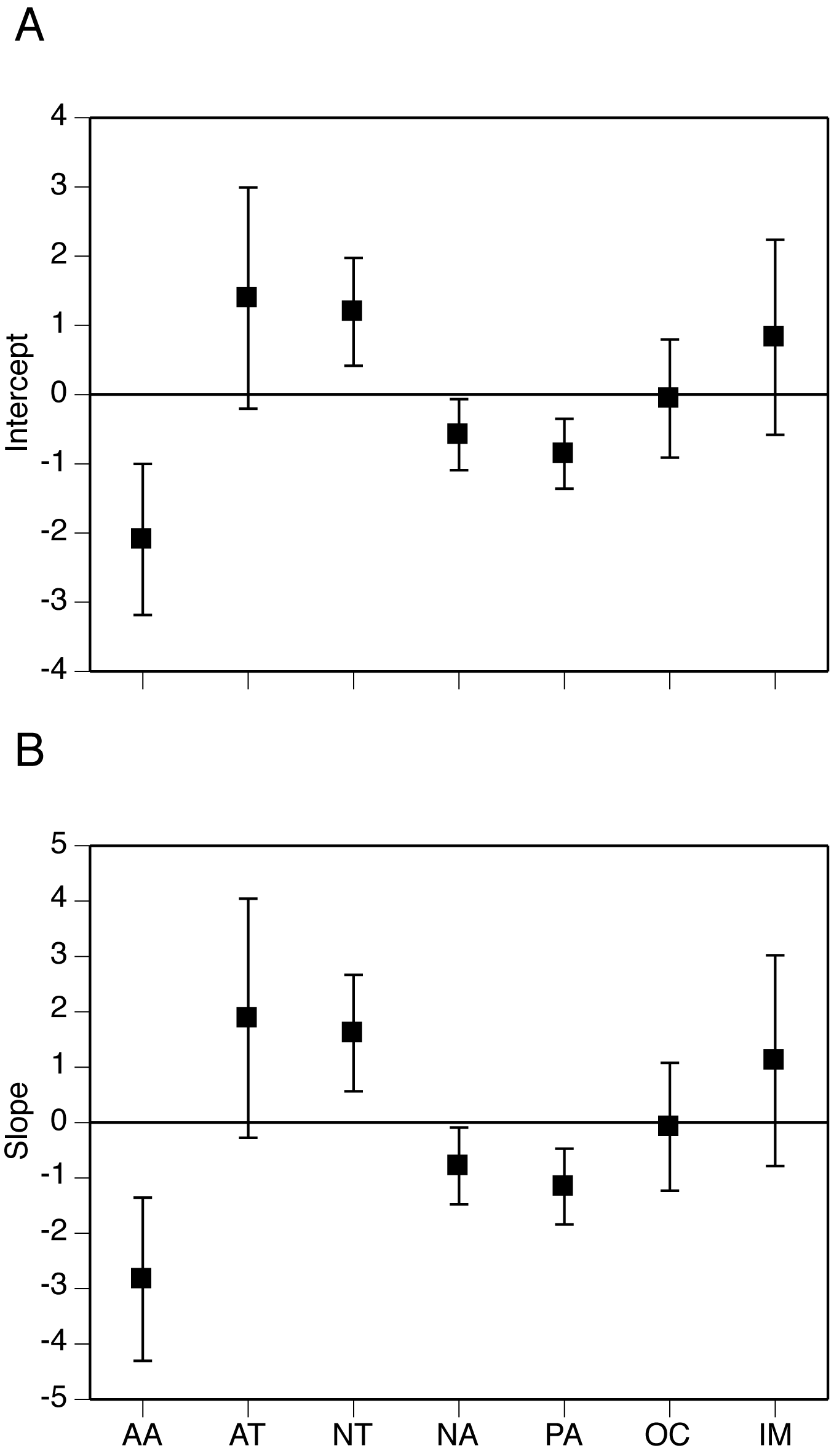

Supplement: Figure S1 — 95% prediction intervals on the conditional modes of the random intercepts and slopes of the relationship between residual brain mass and establishment probability in amphibian and reptile species. (TIF) [file pone.0018277.s001.tif]

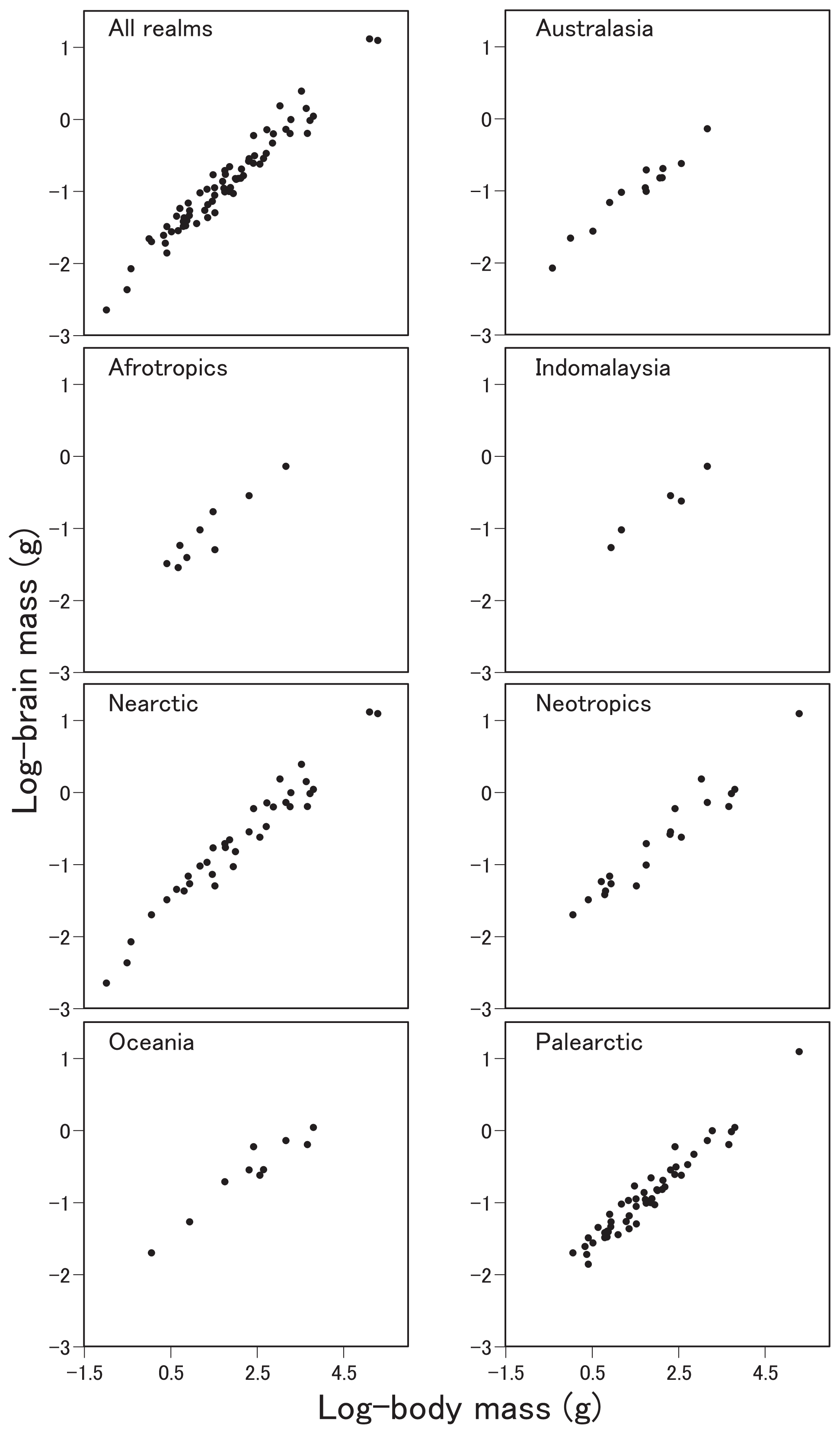

Supplement: Figure S2 — Log brain mass versus log body mass for all amphibian and reptile species used in this study. The brain mass versus body mass trend inclusive of all biogeographic realms is shown in the top left panel, followed by the trends for each individual biogeographic realm. (TIF) [file pone.0018277.s002.tif]
